# Secondary reconstruction of vaginal stenosis using a posterior labial perforator based Falandry flap

**DOI:** 10.11604/pamj.2015.21.185.6559

**Published:** 2015-07-07

**Authors:** Amine Slaoui, Tidiani Kariba Bagayogo, Tarik Karmouni, khalid Elkhader, Abdelatif Koutani, Ahmed Ibn Attaya

**Affiliations:** 1 Urology B, Ibn Sina University Hospital, Rabat, Morocco

**Keywords:** Vaginoplasty, female, reconstruction, Falandry falp

## Abstract

The aim of vaginoplasty should be the creation without excessive morbidity of a neovagina that will be satisfying in appearance, function and feeling. The multitude of methods described in the literature indicates the fact that an ideal approach has not yet been found. In this paper the authors describe the technique for repairing vaginal stenosis by interposing between the vaginal walls, a skin flap pedicled removed using the Falandry technique at a high lip. We achieved a satisfactory result.

## Introduction

**Figure 1:**
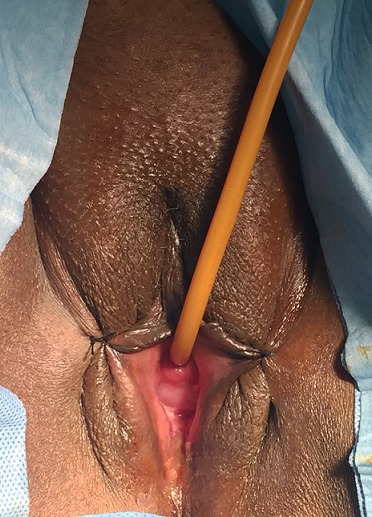
Preoperative aspect: vaginal stenosis

**Figure 2:**
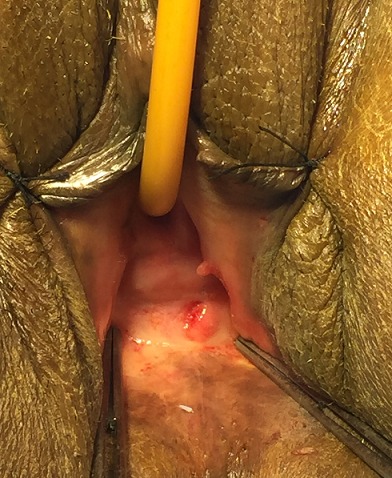
Close view of the vaginal stenosis

**Figure 3:**
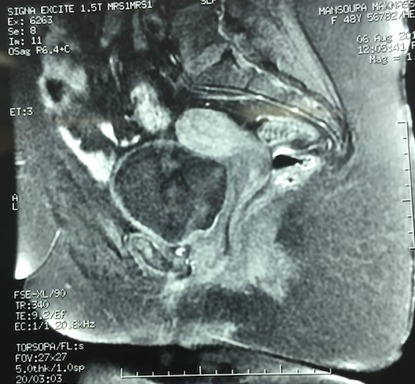
Pelvic MRI (T1): vaginal narrowing

**Figure 4:**
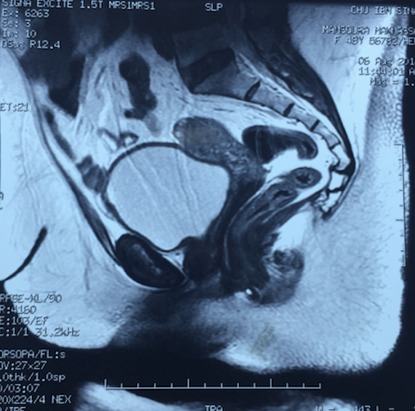
Pelvic MRI (T2): vaginal narrowing

The (re-) construction of a competent vagina in female patients may be indicated in cases where there is absence of a functional vagina. This absence may be absolute, for instance in congenital or iatrogenic cases, or relative, for example after trauma or infection [1]. Various procedures have been applied [2, 3]. The number and variety of these methods indicate that it is a challenging problem, and that the ideal solution to this problem is yet to be established. In this paper we present a case of a 49-year-old woman who had external genital abnormalities due to complication of six pregnancies. She had two operations (without plasty) in another hospital, and this had resulted in severe constriction of the all vagina (Figure 1, Figure 2). Clinically the patient reported dyspareunia complicated by an inability to have sex and therefore conflicts in the family. On physical examination the patient has stenosis of the all the vagina until the cervix. This stenosis is almost complete with impassable finger. Pelvic MRI shows a narrowing of the vaginal caliber on its proximal 1/3 (Figure 3, Figure 4).

## Patient and observation

**Figure 5:**
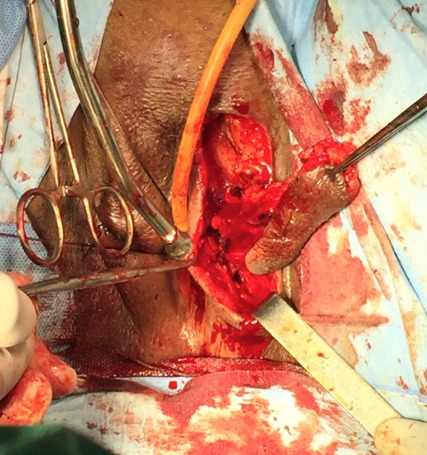
One labial border flap was elevated in advance as local flap

**Figure 6:**
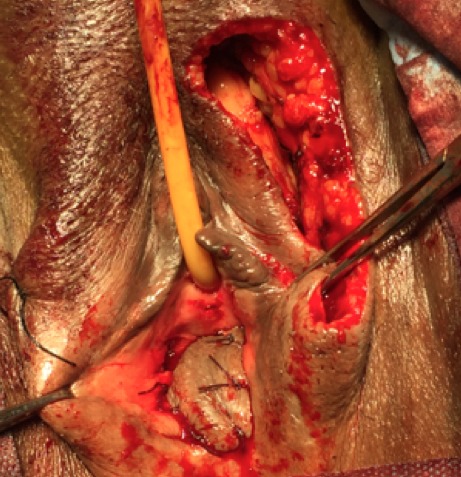
The flap was appointed to form the posterior wall

**Figure 7:**
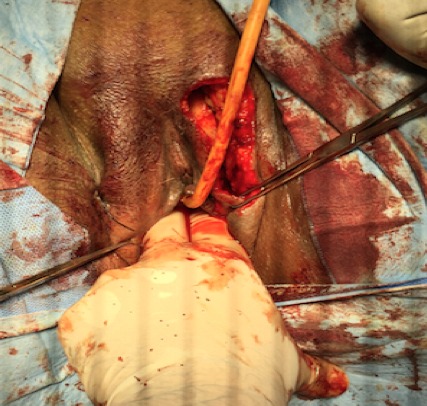
Vaginal lumen of adequate size: introduction of two fingers

The first vaginoplasty using skin grafts was in 1898 Abbe [4] used a rubber pouch covered with a skin graft and stuffed with iodoform gauze to create a neovagina. In 1938 Mclndoe and Banister [5] re-introduced and popularized this technique. The technique we used was repairing vaginal stenosis by interposing between the vaginal walls, a skin flap pedicled removed using the Falandry technique at a high lip [6]. The patient is under spinal anesthesia. Perforators from the posterior labial artery were mapped at the lateral base of the labium major. One labial flap was designed based on those perforators. After a skin incision, one labial border flap was elevated in advance as local flaps, and turned backwards (Figure 5). The flap was appointed to form the posterior wall, creating a vaginal lumen of adequate size (Figure 6, Figure 7). The patient was re-examined three weeks after the surgical act, it is satisfied sexually. After a decline of 3 months, there has been no recurrence.

## Discussion

Various flaps have been developed to overcome the drawbacks of skin grafts for the reconstruction of vagina. These include traditional muscle flaps, such as gracillis muscle flap, transverse abdominal muscle flap, and tensor fascia lata flap, and fascia cutaneous flaps, such as medial thigh flaps and gluteal flaps [7]. However, these conventional flaps have several disadvantages. The pedicle is remote from the perineal region and the flaps lack mobility. Therefore, a large flap is required to cover the defect as well as to provide mobility, which leads to an excessive flap volume and severe donor site scarring. Accordingly, a local flap based on perforators is easier to handle and suit- able for resurfacing. The arterial supply to the perineum is abundant, and arises from vessels of the lower abdomen, medial thigh, and gluteal region [8]. The perforators in the perineum include perforators of the superficial external pudendal artery, anterior cutaneous branch of the obturator artery, and the lateral branch of the posterior scrotal (labial) artery. Networks of chain-type vascular anastomose are formed by these perforators [9]. Because of their abundant blood supply, proximity to the defect and similarity of tissue characteristics, several perineal flaps have been suggested [10, 11]. The perforator from the posterior labial artery originates from the posterior labial artery within 1.5 cm of the posterior margin of the labium, and extends antero-laterally through deep fascia to the posterior-lateral base of the labia major. It gives off at least three to five branches and supplies the inferior perineal regions [8]. The posterior labial artery perforator is reliable and the flap can be elevated safely as long as it is connected to the perforator, allowing the flap to move without tension. Based on the posterior labial artery perforator, the labia is unfolded and the inner and outer surface of the labium major is expanded to form a large surface. Although the size of the flap would appear to be quite inadequate compared to large conventional flaps, its thinness and pliability with a large arc of rotation make it especially suitable for resurfacing the inner lining of the vagina. The design of the flap involves only the labium major, which have soft and elastic tissue appropriate to a vagina. Therefore, an effective outcome can be achieved without further operation. The labial border flap is turned back and closed primarily to the donor site, keeping the natural shape of the labial border and covering the reconstructed vaginal orifice circumferentially. Finally donor site morbidity is minimal [10], and scars are also easily concealed after the reconstruction, permitting a favorable aesthetic outcome. A few reports have described effective treatments for secondary or recurrent stenosis of the vaginal orifice [11]. The labia major has enough lining tissue and skin for this purpose if the flap is designed and transposed based on perforators near the base of the labia major.

## Conclusion

The aim of vaginoplasty should be the creation of a neo-vagina that will be satisfying in appearance, function and feeling [12]. Further, it should not require major and risky surgical intervention, and it should not create new lesions and malfunctions or require long and distressing postoperative treatment [13]. Secondary reconstruction vaginal stenosis using a posterior lip flap perforator based Falandry responds perfectly to these criteria and seems to be an ideal technique.
