# Erratum: Secondary reconstruction of vaginal stenosis using a posterior labial perforator based Falandry flap

**DOI:** 10.11604/pamj.2016.24.150.9193

**Published:** 2016-06-21

**Authors:** The Pan African Medical Journal

**Affiliations:** 1 The Pan African Medical Journal, Kampala, Uganda

**Keywords:** Erratum, vaginoplasty, female, reconstruction, Falandry falp

## Abstract

This erratum corrects article: “Secondary reconstruction of vaginal stenosis using a posterior labial perforator based Falandry flap”, The Pan African Medical Journal. 2015;21:185. doi:10.11604/pamj.2015.21.185.6559.

## Erratum

The original version of this article [1] was corrected. The affiliation of all the authors including the corresponding author has been changed to: Urology B, Ibn Sina, University Hospital, Mohammed V University, Rabat, Morocco. This has been corrected in both the PDF and HTML versions of the article.
